# Microbiome species diversity and seasonal stability of two temperate marine sponges *Hymeniacidon perlevis* and *Suberites massa*

**DOI:** 10.1186/s40793-023-00508-7

**Published:** 2023-06-08

**Authors:** Claire E. Lamb, Joy E. M. Watts

**Affiliations:** 1 Centre for Enzyme Innovation, Portsmouth, UK; 2 School of Biological Sciences, University of Portsmouth, Portsmouth, UK

**Keywords:** Marine sponge, Sponge microbiome, Metataxonomic, Illumina sequencing, Seasonal stability

## Abstract

**Background:**

Marine sponges are diverse and functionally important members of marine benthic systems, well known to harbour complex and abundant symbiotic microorganisms as part of their species-specific microbiome. Changes in the sponge microbiome have previously been observed in relation to natural environmental changes, including nutrient availability, temperature and light. With global climate change altering seasonal temperatures, this study aims to better understand the potential effects of natural seasonal fluctuations on the composition and functions of the sponge microbiome.

**Results:**

Metataxonomic sequencing of two marine sponge species native to the U.K. (*Hymeniacidon perlevis and Suberites massa*) was performed at two different seasonal temperatures from the same estuary. A host-specific microbiome was observed in each species between both seasons. Detected diversity within *S. massa* was dominated by one family, *Terasakiellaceae*, with remaining dominant families also being detected in the associated seawater. *H. perlevis* demonstrated sponge specific bacterial families including aforementioned *Terasakiellaceae* as well as *Sphingomonadaceae* and *Leptospiraceae* with further sponge enriched families present.

**Conclusions:**

To our knowledge, these results describe for the first time the microbial diversity of the temperate marine sponge species *H. perlevis* and *S. massa* using next generation sequencing. This analysis detected the presence of core sponge taxa identified in each sponge species was not changed by seasonal temperature alterations, however, there were shifts observed in overall community composition due to fluctuations in less abundant taxa, demonstrating that microbiome stability across seasons is likely to be host species specific.

**Supplementary Information:**

The online version contains supplementary material available at 10.1186/s40793-023-00508-7.

## Background

Marine sponges have developed close associations with microorganisms, harbouring large quantities of complex symbiotic microbial communities including archaea, algae, cyanobacteria and diverse heterotrophic bacteria [1]. Microorganisms can make up around 40% of the sponge biomass, two times higher than microbial numbers in seawater [2], often differing markedly from associated benthic microbial communities [3]. As sessile filter feeders [4] marine sponges are in constant interaction with the surrounding seawater however, sponges have been shown to harbour distinct sponge associated bacterial communities which are often linked to sponge specific-functions [5]. Dominant sponge associated taxa include the phyla *Proteobacteria, Chloroflexi, Acidobacteria, Cyanobacteria* and candidate phylum *Poribacteria* [6] which can be undetected in or present at low abundances in seawater [7] and previous work has also shown that marine sponges can contain unique bacterial populations that are highly abundant within only one specific sponge species [8, 9]. Whilst others have been shown to more closely resemble the community composition in the surrounding seawater with taxa which are phylogenetically similar to seawater communities and no species-specific relationships being detected [8, 10].

The majority of work characterising the sponge microbiome has been limited to the collection of the host species at a single time point [11], therefore, often the resulting information on associated microorganisms is static, not reflecting any potential shifts or changes within the microbiome [12]. There are numerous environmental changes likely to affect sponge communities including temperature changes [13] as well as differing ecological pressures. Species demonstrating close associations with the associated seawater due to abiotic factors such as location or biotic factors such as internal sponge morphology and high seawater filtering rate [14] may be affected on a microbial community composition level by changes in the surrounding environment. Conversely, other species which are comparatively less closely associated with the surrounding water column (due to differing ecological constraints e.g. the sponge location in the subtidal or intertidal zone), may be affected differently [15].

Changes in the sponge microbiome have previously been assessed in relation to temperature-related changes for seasonal and extreme temperature fluctuations and also other abiotic factors [16–22], some demonstrating high levels of stability [19, 20], and others wherein shifts and disruptions have been observed within the associated microbial communities [16–17, 21–22]. Even minor and naturally occurring variations in environmental conditions can markedly affect organisms [23] which has been demonstrated in tropical, temperate and cold-water sponge species [16–22]. The sponges examined have different preferred environmental niches, although both are submerged at high tide and exposed at low tide *S. massa* is found in deeper, fast flowing water areas [24] and *H. perlevis* found in shallow, slower flowing water [25] in the intertidal zone of the Bosham estuary and Chichester Harbour. In order to better understand the resilience of the sponge microbiome and its complexity, assessing how these communities change over time with differing environmental conditions is key. To fulfil this aim, this present study examines the effect of seasonal changes in temperate sponges located on the South Coast of the U.K.

## Methods

### Sampling and sample processing

Non-fatal samples of five sponges of each two species (*Hymeniacidon perlevis, Suberites massa*) were collected along a transect from the intertidal zone at Bosham Harbour (50.8290° N, 0.8577° W) with 1 L of sea water samples from each sponge collection site using sterile sodium thiosulphate (20 mg/L) bottles (VWR). Sampling was undertaken during two timepoints: July-August (ambient temperature 22 °C, water temperature 22–24 °C, pH 6) (these samples are subsequently referred to as T24 or T22 for temperature 22–24 °C) and October-November (ambient temperature 14 °C, water temperature 15 °C, pH 6) (these samples are subsequently referred to as T15 for temperature 15 °C) with five sponge samples taken at each timepoint, along with 1 L of seawater associated with each replicate sampling site at each individual timepoint collected ≤ 1 m from each sponge sampling site. Due to the tidal variation sample collection was carried out at water depths between 0.02 and 0.9 m. Sponge and seawater samples were collected aseptically and immediately transported to the laboratory for analysis. Sponge samples were rinsed in sterile artificial seawater (ASW) [26], debris was removed, followed by a further wash step with sterile ASW and a representative fragment of tissue (ensuring a complete cross-section of the specimen) was cut using a sterile scalpel. Resulting dissected samples were stored at -80 °C. Seawater samples (1 L) were filtered through cellulose nitrate filters (0.22 μm) (Sigma) and filters stored at -80 °C. Sterile dH_2_0 was filtered as previously described to act as a negative control for further sequencing analysis.

### DNA extraction

For next generation sequencing DNA was extracted from homogenised sponge samples and water filters using QIAamp PowerFecal Pro DNA kit (Qiagen) according to the manufacturer’s protocol. An additional incubation step was added prior to homogenisation in order to increase DNA yields: samples were incubated at 65 °C for 10 min (inverted by hand three times after 5 min) and transferred onto ice. The homogenisation protocol was altered to a speed setting of 4 for 30 s using FastPrep-24 5G homogeniser (MP Biomedicals), pause on ice 5 min and homogenisation repeated as before.

### Sponge species identification

In order to allow visualisation of spicules, samples were prepared [27] and scanning electron microscopy (SEM) analysis was carried out using Zeiss digital scanning electron microscope (Zeiss Evo LS25). Initial visualisation of spicules confirmed sponge samples collected were different species with one species demonstrating smaller microxea spicules (Figure S1A,B) in comparison to the second species with comparatively larger microtylostyle spicules (Figure S1C,D) [28]. Sanger sequencing of the 28S rRNA gene (Eurofins Genomics, Ebersberg, Germany) was carried out with primers C2 (GAAAAGAACTTTGRARAGAGAGT) and D2 (TCCGTGTTTCAAGACGGG) [29] and sponge species were identified as *Hymeniacidon perlevis* and *Suberites massa* following BLAST analysis (sharing 97.43% and 97.18% with accessions MF685334.1 and HQ379249.1 respectively). The raw sequence files were deposited in the Sequence Read Archive NCBI repository under BioProject ID PRJNA922550.

### 16S rRNA gene sequencing

The V3-V4 region of the bacterial 16S rRNA gene was amplified from genomic DNA extracted from *Hymeniacidon perlevis, Suberites massa* and associated seawater at each seasonal temperature point. Next generation sequencing was carried out by LGC Genomics (Berlin, Germany) using the Illumina MiSeq V3 platform. DNA (25 µL) of concentration 1–10 ng/ µL was amplified using bacterial primers 341F (CCTACGGGNGGCWGCAG) and 785R (GACTACHVGGGTATCTAAKCC) [30]. Negative controls (with no sample added) were used for both PCR amplification and Illumina sequencing in order to assess presence of contamination. The raw sequence datasets were deposited in the Sequence Read Archive NCBI repository under BioProject ID PRJNA922550.

### Taxonomic and statistical analysis

Primers and barcodes were removed from demultiplexed raw FASTQ files using Cutadapt v 4.2 [31] in Python v 3.8.15. Sequence quality was assessed using DADA2 [32] in R Studio 1.4.2 v 4.2.2. Forward and reverse quality was plotted and filtered to 260 and 240 respectively. DADA2 was used to trim and filter sequences, predict and correct Illumina sequencing error rates and merge sequences. Samples were individually checked for chimeras using the removeBimeraDenovo function and identified chimeric sequences were subsequently removed from the dataset. Input, filtered, denoised, merged and non-chimeric sequences were tracked throughout the pipeline. Sequences were grouped into features based on 100% sequence similarity, subsequently referred to as ASV (amplicon sequence variants). Taxonomic assignments of ASVs remaining after above filtering steps were classified using SILVA v123 99% Operational Taxonomic Units. Non-bacterial and archaeal (including Chloroplast and Mitochondria) derived sequence reads and singletons were removed from the dataset, low prevalence taxa were filtered by removing ASVs of a low abundance (with a minimum prevalence of 2 ASVs) and the feature table was rarefied to an even sequencing depth (18,606 reads from *H. perlevis* and *S. massa*, 9,096 reads from *H. perlevis* and associated seawater and 16,664 reads from *S. massa* and associated seawater).

All diversity and statistical analyses were performed in R Studio 1.4.2 v 4.2.2 using vegan, phyloseq and microeco packages [33–35]. Following rarefaction, the richness of samples was analysed using observed diversity and alpha diversity indices (Shannon index, Simpson index). In order to determine significance of differences between alpha diversity indices Kruskal-Wallis one way ANOVA and Dunn’s Kruskal-Wallis Multiple Comparisons test was carried out in R Studio 1.4.2 v 4.2.2. Principal component analysis (PCoA) based upon Bray Curtis distance metrics was carried out in order to assess the similarities and dissimilarities between samples and within groups. Permutational multivariate analysis of variance (PERMANOVA) (P = ≤ 0.01), [36] was conducted in R package vegan [33] using Bray Curtis similarity matrix from transformed abundance data to test the significance of bacterial community dissimilarities. Core bacterial taxa were determined based on 100% prevalence (present in all replicates) and 0.01% relative abundance [37, 38]. All graphs were created in R Studio 1.4.2 v 4.2.2 using ggplot2, phyloseq and microeco packages [33–35, 39].

## Results

Sponge species were identified as *Hymeniacidon perlevis* and *Suberties massa* following visualisation of spicules and 23 S rRNA sequencing. A total of 2,123,269 Illumina reads were obtained from *H. perlevis* and surrounding seawater of which 1,427,031 remained after quality checks and filtering steps. From *S. massa* and associated seawater 2,538,379 total Illumina reads were obtained after which 1,791,114 remained after quality filtering (Table S1).

Data was subsampled (Figure S2) to the minimum read depth however, a number of samples, such as *H. perlevis* (particularly from 24 °C), had higher numbers of detected ASVs (ASV curves have not reached a plateau at the minimum read depth) and the diversity of these may not be fully represented in subsampled data. Permutational multivariate analysis of variance (PERMANOVA) results (Table S4) show significant differences between all samples aside from between samples of *H. perlevis* from each seasonal timepoint, indicating dissimilarities in bacterial community composition.

### Species-specificity

**Fig. 1:**
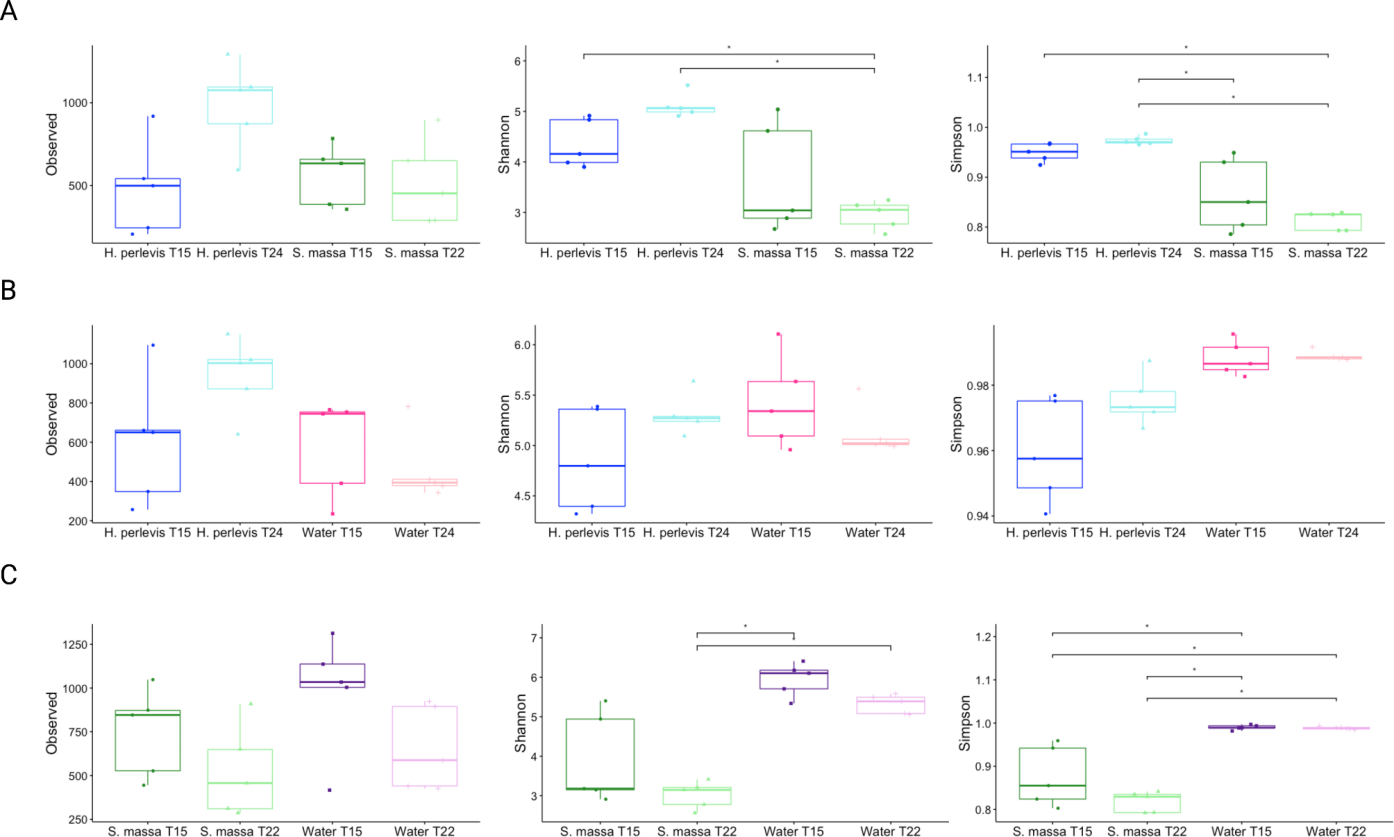
Alpha diversity matrices (observed, Shannon diversity index, Simpson diversity index) for A) H. perlevis and S. massa, B) H. perlevis and associated seawater and C) S. massa and associated seawater. Significant differences between each group are measured by Kruskal-Wallis one-way ANOVA (*= p ≤ 0.05, **= p ≤ 0.01, ns = not significant). Full alpha diversity values and statistics can be found in Supplementary Tables 2–3.

**Fig. 2:**
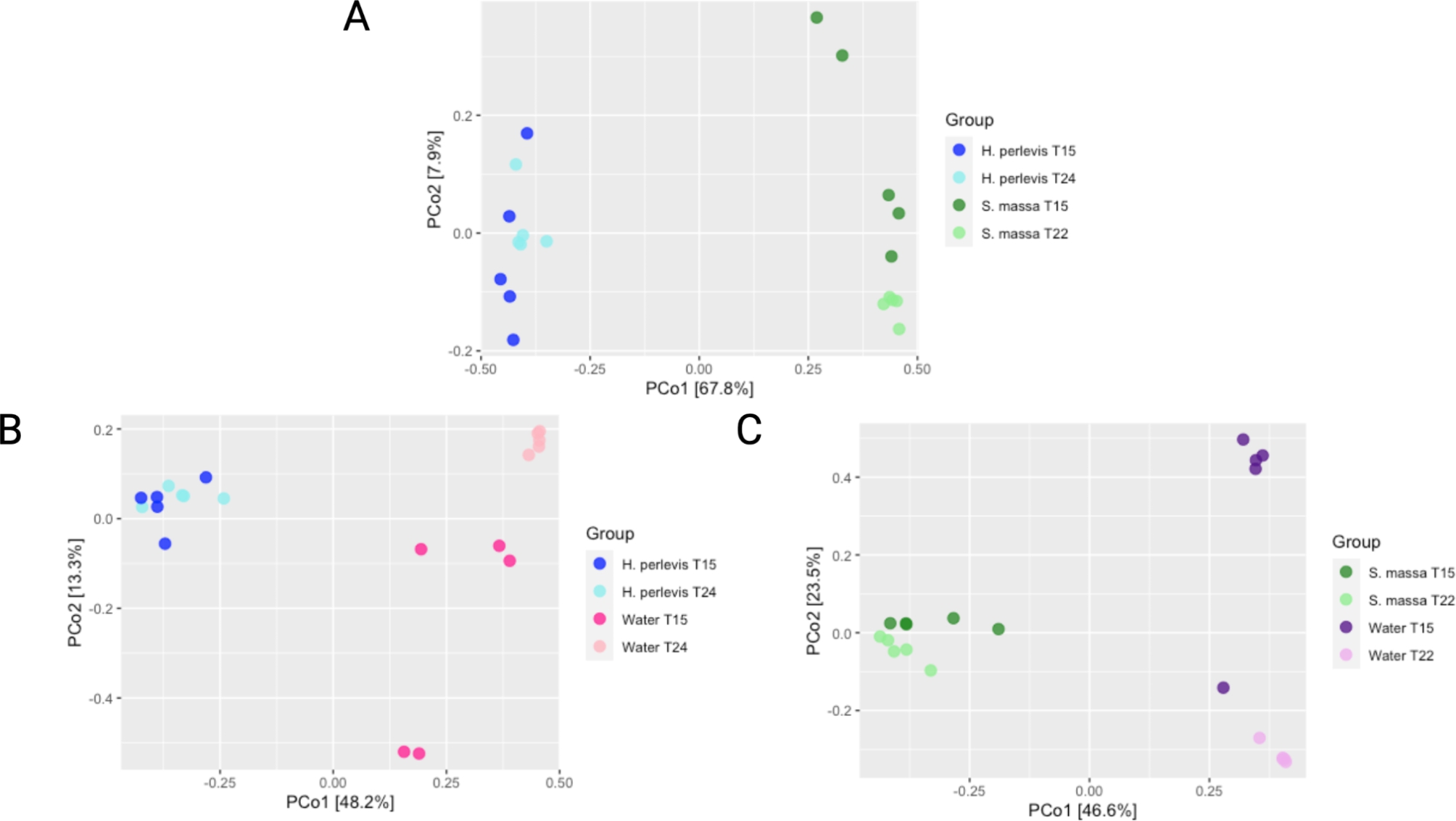
Principal coordinate analysis (PCoA) of A) H. perlevis and S. massa, B) H. perlevis and associated seawater and C) S. massa and associated seawater. X and Y axis’ represent coordinates of the greatest sources of variation within datasets representing A) 7.9% and 67.8% B) 13.3% and 48.2% and C) 23.5% and 46.6% respectively.

When observed and Alpha diversity measures (Shannon index, Simpson index) were applied to analyse diversity within samples (Fig. 1) there were no significant differences in terms of abundance (observed diversity) between samples of *H. perlevis* and *S. massa* however *H. perlevis* samples demonstrated higher Shannon and Simpson diversity indices than *S. massa* samples (Fig. 1A; Table S2; Table S3). Beta diversity assessing diversity between samples show clear differences in diversity clustering and values detected between samples of each sponge, with samples of *H. perlevis* (Fig. 2A; Figure S2A) demonstrating similarities in bacterial composition across samples in comparison to samples of *S. massa* which clearly clustered apart from samples of *H. perlevis* (Fig. 2A).

**Fig. 3:**
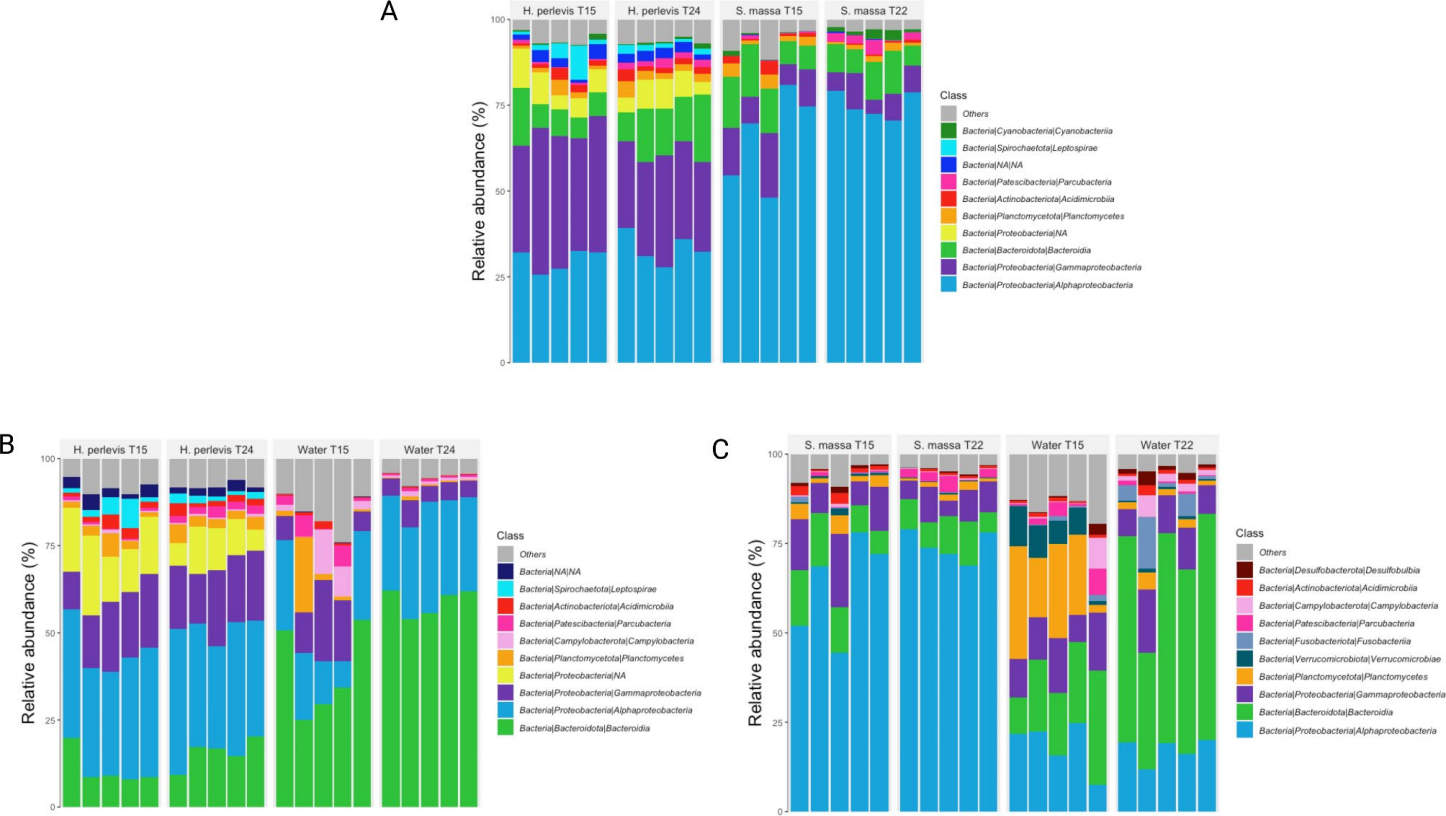
Stacked bar plot of the distribution of bacteria at class taxonomy level showing the top 10 classes in each sample and remaining classes shown in grey A) H. perlevis and S. massa, B) H. perlevis and associated seawater and C) S. massa and associated seawater.

Community composition at phylum level (Table S5) was similar between both sponge hosts, with the majority of diversity in all samples consisting of *Proteobacteria* (67.8–74.6% in *H. perlevis* and 77.2–82.1% in *S. massa*) followed by *Bacteroidiota*, *Planctomycetota* and *Actinobacteriota* though these accounted for less diversity. Samples of *H. perlevis* demonstrated higher detected relative abundance (1.7–3.6%) of the phylum *Spirochaetota* in terms of relative abundance in comparison to *S. massa* (0.4–0.9%) though these differences were not significant (P = 0.12). At class level (Fig. 3A; Table S5) samples of *S. massa* were dominated by *Alphaproteobacteria* (65.6–74.9%) at significantly higher numbers (P = < 0.001) than samples of *H. perlevis* (30-33.3%). The second dominant class *Gammaproteobacteria* comprised a comparatively higher detected relative abundance within *H. perlevis* (28–37%) in comparison to *S. massa* (7.2–11.4%) (P = < 0.001). Both sponge species demonstrated comparable numbers of *Bacteroidia* (8.8–14% in *H. perlevis* and 8.9–11.4% in *S. massa*), *H. perlevis* specific class (undetected within samples of *S. massa*) *Leptospirae* varied from an average of 1.7–3.6% relative abundance within samples of *H. perlevi*s.

**Fig. 4:**
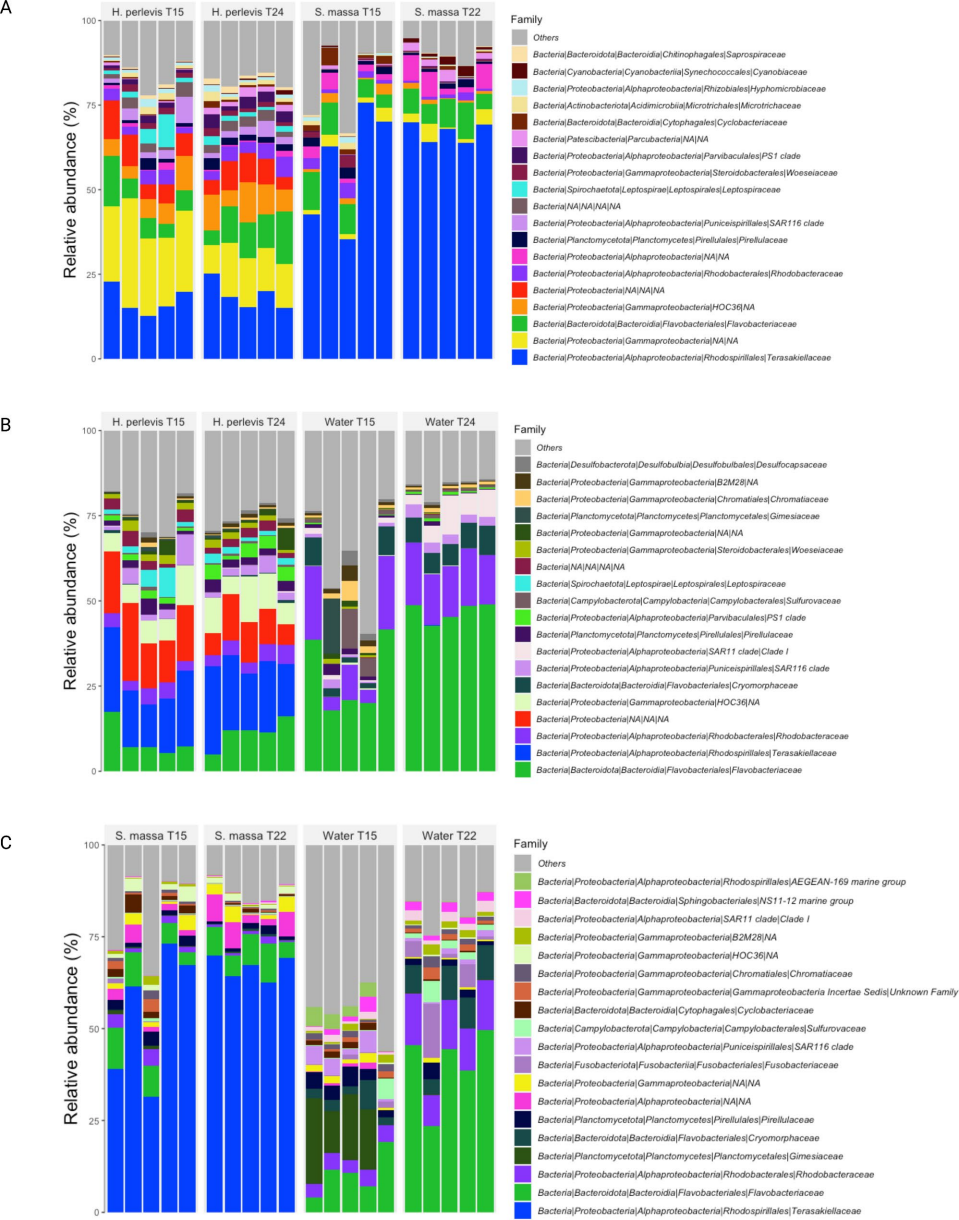
Stacked bar plot bacterial distribution at taxonomic level of family showing the top 20 families in each sample and remaining classes are shown in grey A) H. perlevis and S. massa, B) H. perlevis and associated seawater and C) S. massa and associated seawater.

Despite similarities in diversity detected at phylum and class level (Fig. 3A) *H. perlevis* demonstrated a comparatively more complex microbiome at family level (Fig. 4A) with sponge specific and sponge enriched families present, wherein sponge specific refers to families only detected within the sponge samples which were absent from or undetected in surrounding water samples and sponge enriched refers to families which were also detected in surrounding water samples but accounted for significantly less diversity. Both sponges demonstrated high levels of *Flavobacteriaceae* (*Flavobacteriia*) and *Terasakiellaceae* (*Alphaproteobacteria*) with numbers of *Terasakiellaceae* being significantly higher (P = 0.001) within samples of *S. massa* (57.3–67.2% in *S. massa* in comparison to 17.1–18.6% in *H. perlevis*) (Table S8). *H. perlevis* specific families included SAR 116 (*Alphaproteobacteria*), *Leptospiraceae* (*Spirochaetota*) and *Sphingomonadaceae* (*Alphaproteobacteria*) not being detected within samples of *S. massa*. *S. massa* samples demonstrated higher detected relative abundance of the families *Cyclobacteriaceae* (*Cytophagia*) and *Cyanobiaceae* (*Cyanobacteria*) (P = 0.01, P = 0.05 respectively).

### Microbiome composition

No significant differences were detected in observed diversity, richness/ evenness (Shannon diversity index) or distribution of species when also considering abundance (Simpson’s diversity index) between samples of *H. perlevis* and associated seawater samples (Fig. 1B; Table S2; Table S3). However, similarly to significantly higher detected richness when compared to samples of *H. perlevis*, seawater samples also demonstrated higher Shannon and Simpson values than samples of *S. massa* (Fig. 1C; Table S2; Table S3). Distance values and clusters between samples of *H. perlevis* and associated seawater (Fig. 2B) demonstrated a clear distinction between sponge and water samples, with both *H. perlevis* and *S. massa* samples demonstrating a distinct microbiome composition both from their associated seawater samples and from the other sponge host, with intra-species samples clustering closely together (Fig. 2B and C; Figure S2B; Figure S2C). Furthermore, water samples were grouped together, albeit with more variability shown between samples of seawater taken at 15 °C.

When comparing the sponge communities to the surrounding water column *H. perlevis* was dominated by *Proteobacteria* (*Alphaprotebacteria* followed by *Gammaproteobacteria*), and *Bacteroidiota* (*Bacteroidia*) which accounted for the majority of relative abundance in each of the *H. perlevis* samples (Fig. 3B; Table S6) and were the same dominant phyla and classes observed in seawater samples. Dominant families which were detected in *H. perlevis* include *Flavobacteriaceae (Flavobacteriia*), *Rhodobacteraceae (Alphaproteobacteria)* and *Terasakiellaceae (Alphaproteobacteria)* (Fig. 4B; Table S8). In comparison the community composition in surrounding seawater consisted of significantly higher numbers of *Flavobacteriaceae* (P = 0.01) and *Rhodobacteraceae* (P = 0.003) as well as *Cryomorphaceae (Flavobacteriia)* (P = 0.009) with variation between samples of seawater taken at 15 °C. Sponge specific families included *Leptospiraceae* (*Spirochaetia*), *Sphingomonadaceae* (*Alphaproteobacteria*) and an unidentified family of the order *Dadabacteriales* which were associated only with the sponge host and not detected in the surrounding seawater (Table S9).

*S. massa* samples were also shown to contain a high proportion of *Proteobacteria* (predominantly *Alphaproteobacteria* accounting for 63-74.3% of the detected relative abundance) and *Bacteroidiota* (*Bacteroidia* compromised a mean of 8.8% detected relative abundance), which were the same dominant phyla and classes in seawater samples (Fig. 3C). *Proteobacteria*, *Bacteroidiota*, and *Planctomycetota* were the three dominant phyla in water samples however seawater replicates show variation between timepoints (Fig. 4C; Table S6).

The sponge specific family *Terasakiellaceae* (*Alphaproteobacteria*) accounts for the majority of the detected relative abundance in *S. massa* samples (54.9–66.6%) followed by *Flavobacteriaceae (Flavobacteriia)* (7.2–7.6%) (Fig. 4C; Table S9), also detected in seawater samples. Sponge samples also contained sponge enriched families *Cyclobacteriaceae (Cytophagia)* and *Cyanobiaceae* (*Cyanobacteria*), these were detected at significantly higher numbers than in seawater (P = 0.009 and P = 0.002 respectively). Seawater samples were generally dominated by *Flavobacteriaceae* and *Rhodobacteraceae (Alphaproteobacteria)*, with these families also being present in *S. massa* samples, but in a lower proportion than in seawater samples (Fig. 4C; Table S10) with significantly fewer counts of *Rhodobacteraceae* (P = 0.01).

### Seasonal stability

Whilst there were no significant differences detected in observed diversity, evenness of species (Shannon index) or distribution of species considering abundance (Simpson’s index) (Fig. 1A). Samples taken between seasons show no clear separation for samples of *H. perlevis* (Fig. 2A) indicating comparable bacteria community compositions. At phylum level, the same five phyla remained dominant *Proteobacteria, Bacteroidiota, Planctomycetota, Actinobacteriota* and *Spirochaetota* (Table S5). However, in *H. perlevis* samples taken at 15 °C the average relative abundances for *Gammaprotebacteria* (*Proteobacteria*) and *Leptospirae* (*Spirochaetota)* increased from 28 to 37% and 1.7–3.6% respectively (P = 0.01, P = 0.3 respectively) with *Parcubacteria* (*Pastescibacteria*) falling from 2% to 24 °C to 0.6% at 15 °C (P = 0.001). At family level no significant differences were observed in relative abundance between dominant families associated with *H. perlevis* samples at both seasons (Table S8). Additionally, no changes in detected relative abundance of core bacterial taxa were observed at each season with a total of 69.3% detected relative abundance (made up of 807 ASVs) being shared between *H. perlevis* in both seasons (Figure S4B).

No significant differences were observed in abundance of species, species richness or evenness between samples of *S. massa* at different seasonal temperatures (Fig. 1A). However, whilst replicates of samples cluster closely, there is a separation (Fig. 2A) between those taken in the warmer season (22 °C) and those taken in the cooler season (15 °C) suggesting inter-seasonal differences in community composition (P = 0.008) with those taken at the colder temperature (15 °C) showing more variability between replicates in comparison to the warmer temperature (22 °C). The same three classes remained dominant *Alphaproteobacteria, Bacteroidia* and *Gammaproteobacteria* (Table S5) with both *Gammaproteobacteria* and *Bacteroidia* increasing in terms of relative abundance in the cooler (15 °C) season (from 7.2 to 11.4% and 8.9–11.4% respectively) and *Alphaproteobacteria* decreasing (from 74.9 to 65.6%), although none of these differences were statistically significant. At family level *Terasakiellaceae* and *Flavobacteriaceae* remained dominant with non-significant differences between seasons (Table S9). Shifts in proportions of family *Cyanobiaceae* were observed demonstrating higher numbers (from 1.7 to 0.2% detected relative abundance) in sponge samples from the warmer (T22°C) time point (P = 0.01). A total of 68.8% of the detected relative abundance (made up of 624 ASVs) being shared between *S. massa* at different seasons (Figure S4C).

## Discussion

To our knowledge, this study presents the first NGS based examination of microbial diversity in the temperate sponges *H.perlevis* and *S.massa*. As previously observed in other sponge species [40, 41] *H. perlevis* and *S. massa* samples were dominated by *Proteobacteria* (*Alpha*- followed by *Gamma*) and *Bacteroidiota* which were also detected within seawater samples. These phyla have previously been detected in cold water sponges, particularly Antarctic sponges [42, 43], accounting for a large proportion of bacterial relative abundance. With further previous work [44] showing that *Alpha-* and *Gamma-proteobacteria* account for high amounts of culturable diversity within *H. perlevis*.

In *S. massa* the majority of detected relative abundance was accounted for by one family (*Terasakiellaceae*), which was also detected in samples of *H. perlevis* but as a smaller proportion of the overall observed population. This family, which in both species, is sponge-specific has previously been associated with marine sponges [45], accounting for similarly large proportion of the sponge microbiome as well as in other marine organisms including coral [46, 47]. While the exact functional role this family play in the sponge is not yet established, they are hypothesised to be involved in nitrogen cycling [46], of which marine host associated microbial communities including those of sponges are known to play a key role [48]. As in *S. massa, Terasakiellaceae* accounts for large proportions of bacterial abundance in other marine sponges (*Clathria prolifera* and *Halichondria bowerbanki* from the Mid Atlantic) [44] alongside *Flavobacteriaceae* (also detected in *S. massa* and *H. perlevis*), suggesting these families could be stable members of some sponge microbiomes regardless of sponge species or geographical location.

Differences are apparent in terms of the presence of higher numbers of sponge specific and enriched families within the microbiome of *H. perlevis* in comparison to *S. massa.* The family *Sphingomonadaceae* detected in *H. perlevis* but not in *S. massa*, has previously been demonstrated to be dominant in marine sponge *Rhopaloeides odorabile* [49]. Families of the order *Sphingomondales* are known to form close relationships with marine sponges and have been linked to vitamin B12 synthesis via functional metagenomics [50]. Additionally, the presence species of *Sphingomonadaceae* have previously been demonstrated to enhance degradation rates of artificial chemicals such as Bisphenol A [51]. As *H. perlevis* has been shown to have a high capacity for bioaccumulation of pollutants [52] this contrasting ecological condition could go some way to explaining the presence of *Sphingomonadaceae* in *H. perlevis* and not *S. massa.* However, previous links to marine sponges and associations with vitamin synthesis [49, 50] may conversely suggest that whilst members of this family do not always account for large amounts of overall abundance their role in the sponge microbiome is important and shared across certain sponge species.

These differences in microbiome composition (i.e. the presence of more sponge enriched and specific families within the microbiome of *H. perlevis*) may be accounted for by differences in sponge morphology, for example size or density of mesohyl tissues and narrower water filtering canals [55] as well as contrasting ecological pressures. Species of *Suberites*, which are primarily located in sub and intertidal zones, have previously been suggested to have their entire nutritional needs met by bacterial uptake [56]. This is probably due to a high filtering rate [57] which is likely to have an effect upon both the sponge host and the resident microbial communities. *H. perlevis*, however, is primarily located in rocky, intertidal areas often semi enclosed/enclosed waters and polluted areas, such as ports and harbours [58, 59] with these contrasts in environmental parameters being likely to have an impact upon community composition.

Further contrasting ecological parameters including temperature have been shown to lead to variations in sponge symbionts irrespective of location [60] and may affect, or even determine the symbiotic community with the ability to quickly acclimatise to environmental change essential to sessile organisms [4]. Variations and shifts in environmental conditions can have marked effects on organisms and their physiology [14] and thus can have varying impacts on the associated microbiome which has previously been demonstrated in tropical and temperate sponge species [16, 17, 21, 22]. Previous work has assessed the stability of various sponge microbiomes across geographical and seasonal changes, for example, in reefs off the coast of Florida [61] wherein slight shifts in bacterial taxa were observed including changes in the numbers of *Alphaproteobacteria*, *Gammaproteobacteria* and *Cyanobacteria* across seasons, similarly to both *H. perlevis* and *S. massa* (although the differences in these numbers were not significant). When assessing seasonal stability from spring to autumn of sponge species the Caribbean Sea [62] significant differences were detected between microbial community structure at each season. High levels of variability in the same sponge species across seasons has been observed [12, 13] and this variability has also been demonstrated in other species of *Hymeniacidon* [63]. Whilst shifts were apparent in bacterial community composition these may be attributed to transient or ‘generalist’ bacterial associations as differences within the dominant microbial diversity at family level between *H. perlevis* samples at each season were not found to be significant in terms of detected relative abundance suggesting stability of dominant core *H. perlevis* taxa (69.3% shared between seasons) irrespective of environmental changes such as temperature or changes in the diversity of the surrounding water column. Despite observed changes in the bacterial diversity of the surrounding water column and seasonal changes as well more variability observed between microbial community similarity in samples taken at the cooler temperature in comparison to the warmer temperature, 68.8% of the detected relative abundance was shared between samples of *S. massa* at each season. As with *H. perlevis*, slight shifts in detected relative abundance of families were not found to be significant, aside from significantly higher numbers of the family *Cyanobiaceae* at the warmer (22 °C). Cyanobacterial sponge symbionts have previously been observed to fluctuate across seasons [61] likely due to changes in light availability. The overall microbial community composition in both sponge species appeared to be stable, with minimal significant differences (aside from aforementioned *Cyanobiaceae* in *S. massa*) in dominant sponge associated families. Differences were observed in bacterial community composition of *S. massa* samples from each season, however core dominant taxa were stable. Detected diversity consisted mainly of sponge-specific *Terasakiellaceae* in both seasonal temperatures with this study suggesting this family to be a stable member of both the *S. massa* and *H. perlevis* microbiomes. Observed shifts in bacterial community composition in samples *of S. massa* between seasonal temperatures, whilst this may be attributed to changes in transient associated bacterial communities or ‘generalist’ populations as well as sponge enriched *Cyanobiaceae*, may also be related to host species or related ecological pressures e.g. close association with surrounding seawater. These results suggest, as per previous work assessing the responses of sponges to varying temperature changes including implications for climate change [64], that the temperature response of sponge microbiomes to seasonal change is likely to be sponge-species-specific as well as dependent upon the extremity of the temperature to which they are exposed.

## Conclusions

Similar to other sponge phyla [40, 41] samples of *H. perlevis* were dominated by *Proteobacteria* (*Alpha*- and *Gamma*-), and *Bacteroidiota* (*Bacteroidia*) accounting for the majority of detected relative abundance. This is similar to cold-water sponges e.g. Antarctic sponges [42, 43]. Differences in species richness and microbiome complexity at family level were observed, with *H. perlevis* demonstrating more sponge enriched and sponge specific communities differing from the community composition of the surrounding seawater. Whereas within *S. massa* the majority of the community was *Proteobacteria* (of this was predominantly *Alphaproteobacteria)*, and consisting of one sponge specific family, *Terasakiellaceae.*

Previous work has demonstrated seasonal variability in cultivable bacterial of other *Hymeniacidon* species [63] as well as shifts in terms of sponge specific symbionts such as *Cyanobacteria* [60]. The microbiome *of H. perlevis* appears to be stable in terms of detected core sponge associated taxa in relation to environmental changes such as seasonal changes. Higher diversity was observed in seawater samples in comparison to the microbial diversity of *S. massa* in either season, with higher Shannon and Simpson diversity indices, changes were observed in overall bacterial community composition between samples of *S. massa* at each season, however core families (including sponge specific *Terasakiellaceae)* remained dominant.

This is the first report of the microbial community associated with both of the temperate marine sponge species *H. perlevis* and *S. massa.* The understanding of the microbial community in this study, supports that the temperate sponge microbiome is species specific, and furthermore, that microbiome changes in response to seasonal temperature change are related to this host-specificity. The core taxa detected in the sponge microbiome remained stable, but fluctuations were more commonly detected in the less abundant microbial taxa. This research further assesses the impacts of natural environmental changes on the sponge holobiont, with implications for environmental health and stability related to the wider benthic functioning with which they are associated with.

## Electronic supplementary material

Below is the link to the electronic supplementary material.

Supplementary Material 1

Supplementary Material 2

Supplementary Material 3

Supplementary Material 4

Supplementary Material 5

## Data Availability

The raw 16 S rRNA supporting the conclusions of this article is available in the Sequence Read Archive in NCBI repository under BioProject ID PRJNA922550, submission ID SUB12524464. The raw 28 S rRNA gene sequences are available under BioProject ID PRJNA922550, accession numbers SAMN33299855-SAMN33299856.
