# Supplementary figures and images for: Microbiome species diversity and seasonal stability of two temperate marine sponges Hymeniacidon perlevis and Suberites massa
Source: Environ Microbiome. 2023 Jun 8;18:52. doi: 10.1186/s40793-023-00508-7 (PMC10251714; doi:10.1186/s40793-023-00508-7)

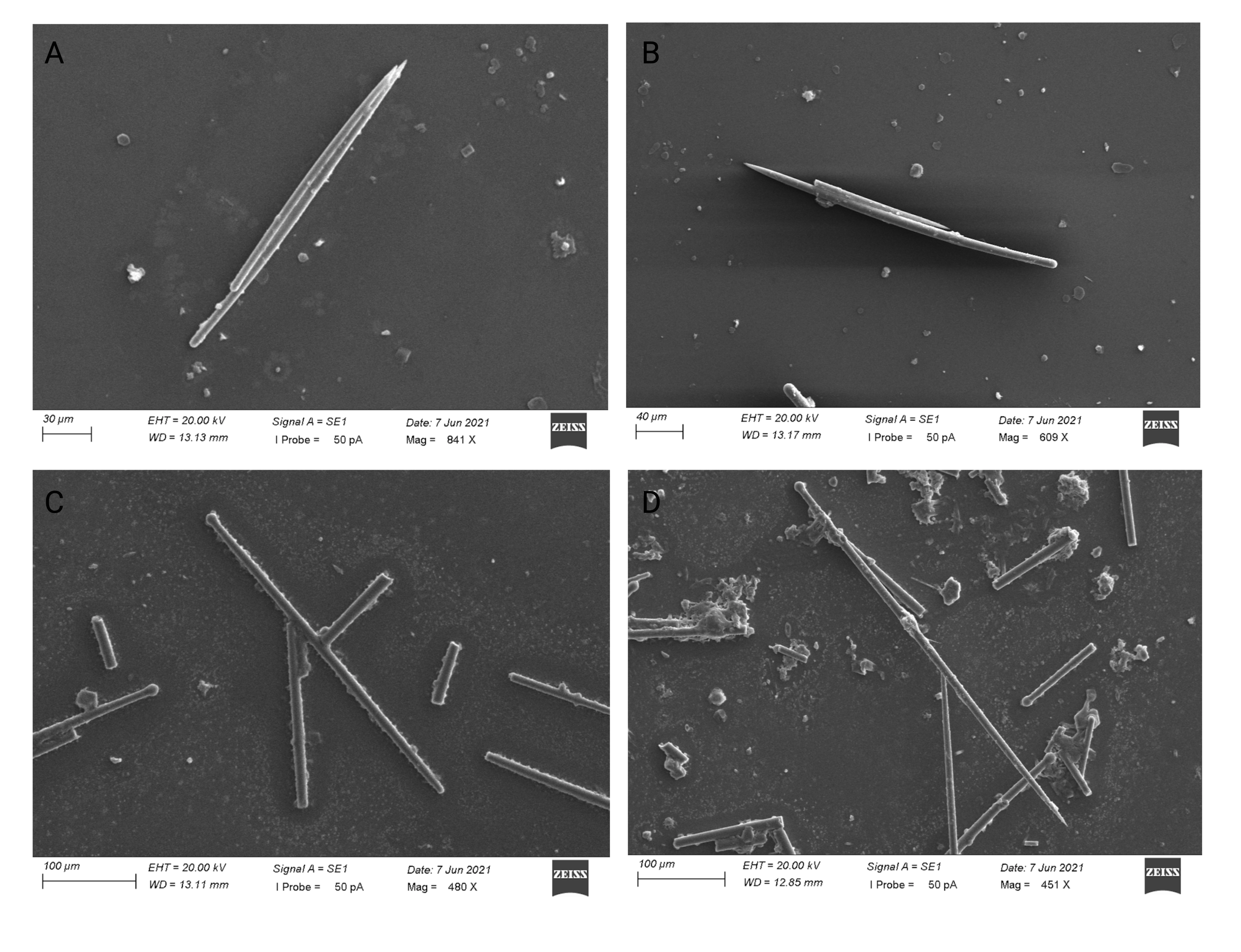

Supplement: Supplementary file 1 — Supplementary Material 1 [file 40793_2023_508_MOESM1_ESM.png]

A

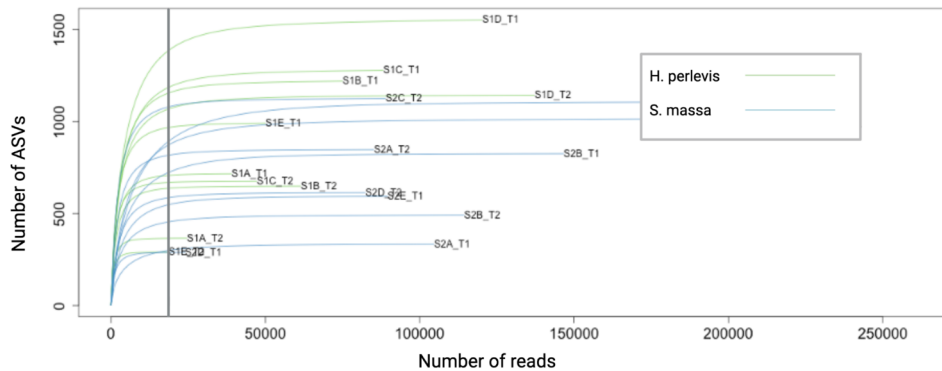

B

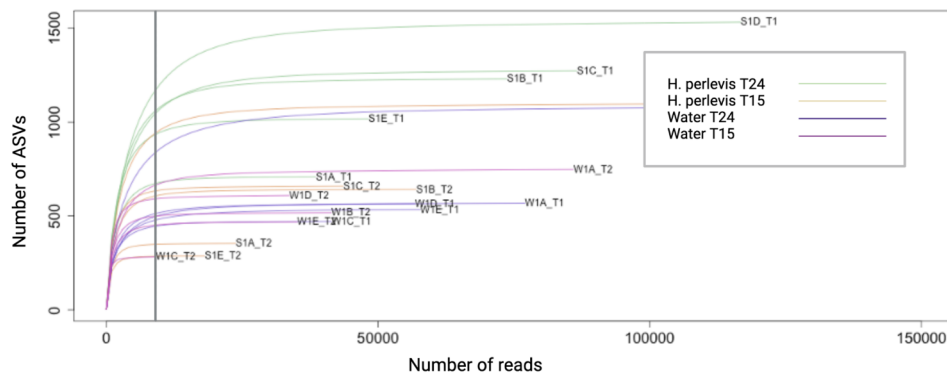

C

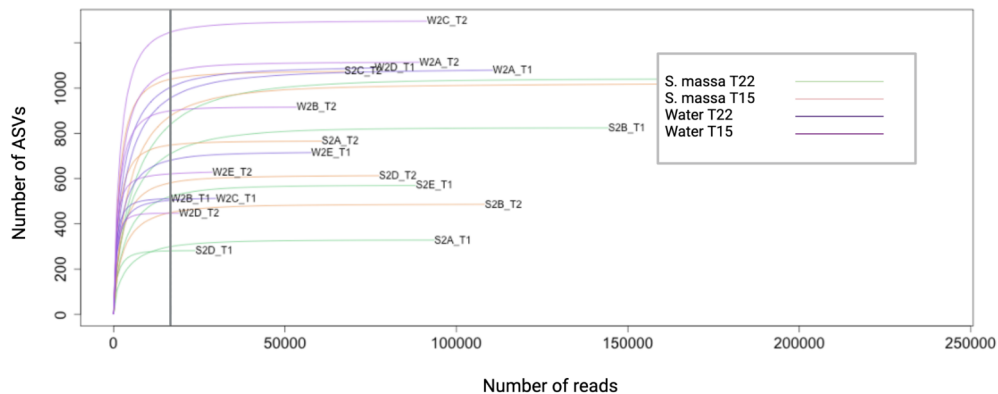

Supplement: Supplementary file 2 — Supplementary Material 2 [file 40793_2023_508_MOESM2_ESM.pdf]

A

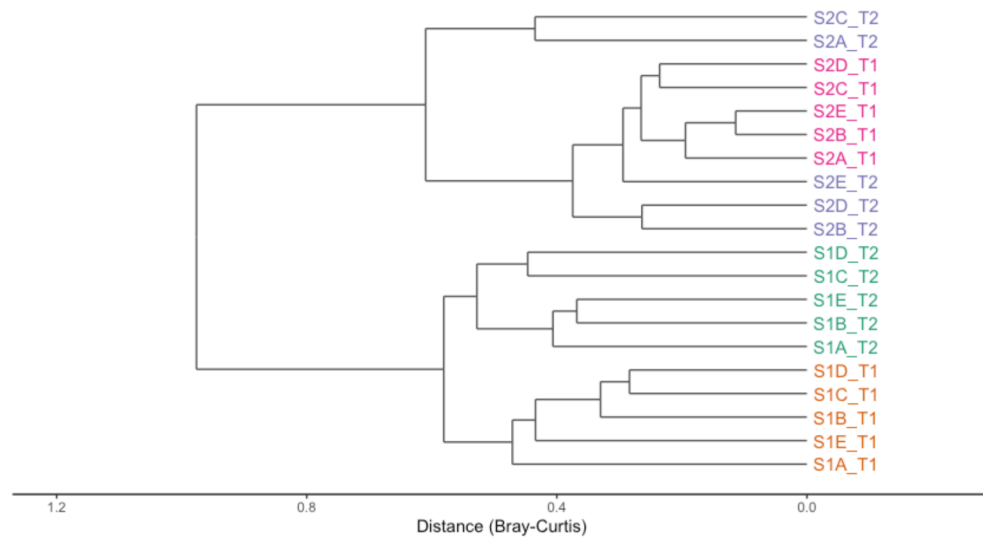

C

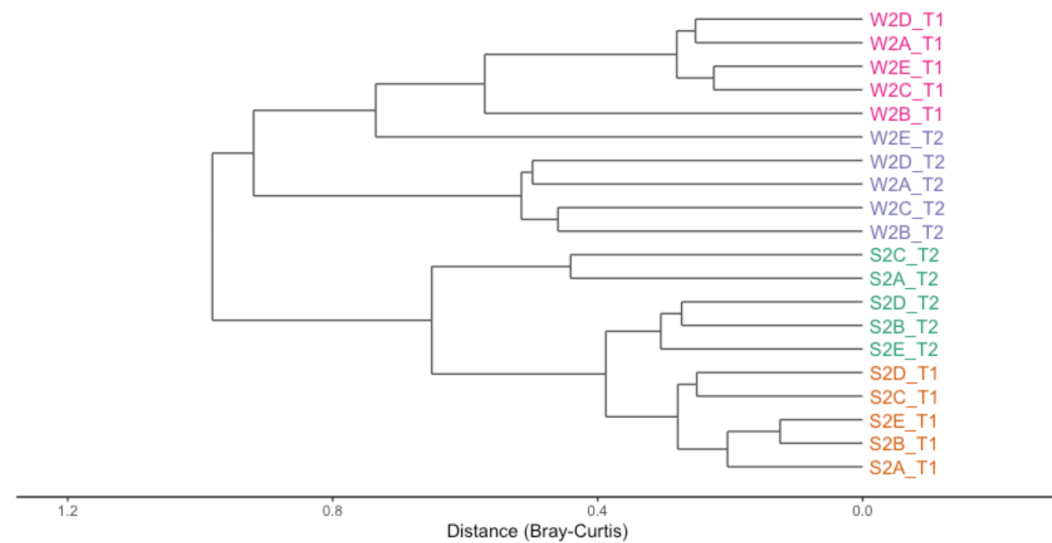

B

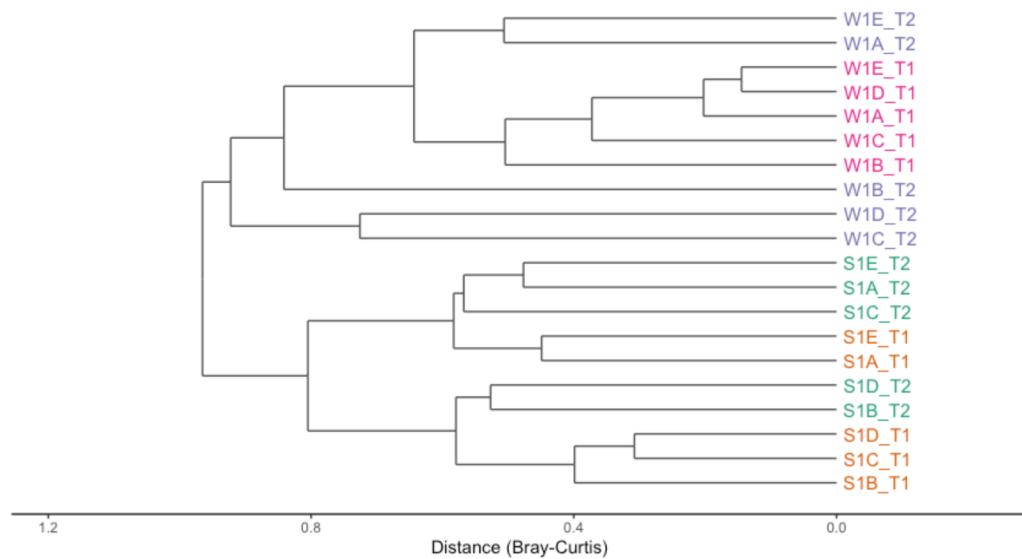

Supplement: Supplementary file 3 — Supplementary Material 3 [file 40793_2023_508_MOESM3_ESM.pdf]

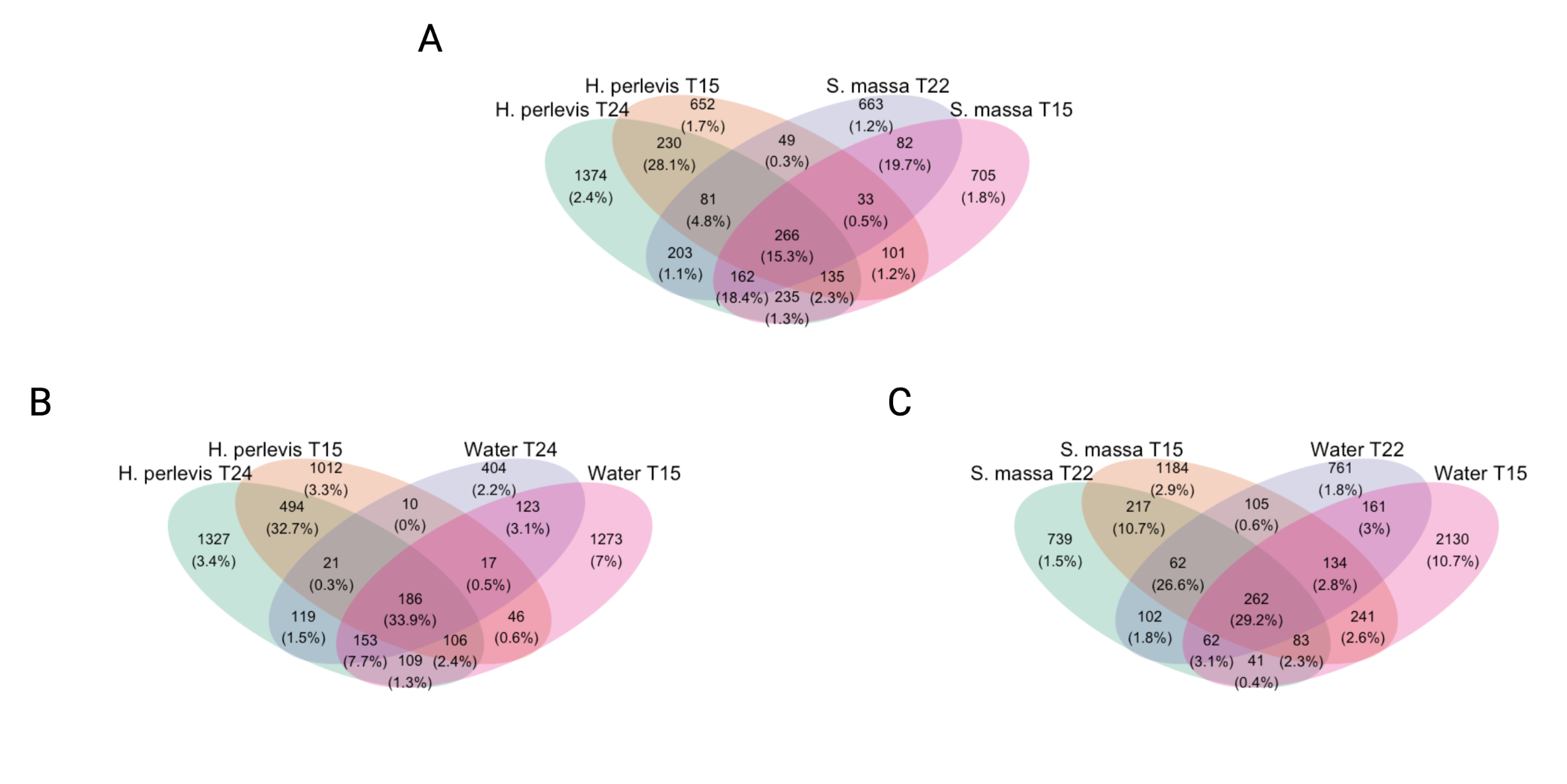

Supplement: Supplementary file 4 — Supplementary Material 4 [file 40793_2023_508_MOESM4_ESM.png]
