# Supplementary material for: Microbiome species diversity and seasonal stability of two temperate marine sponges Hymeniacidon perlevis and Suberites massa
Source: Environ Microbiome. 2023 Jun 8;18:52. doi: 10.1186/s40793-023-00508-7 (PMC10251714; doi:10.1186/s40793-023-00508-7)
Supplement: Supplementary file 5 — Supplementary Material 5 [file 40793_2023_508_MOESM5_ESM.docx]

**Tables**

**Table S1.** Raw input, filtered, denoised and non-chimeric final read counts for *H. perlevis, S. massa*, associated seawater and sequencing negative control (T24= temperature 24 °C, T22= temperature 22 °C, T15= temperature 15 °C)

| Sample | Raw | Filter | Denoised Forward | Denoised Reverse | Merged | Non- chimeric |
| --- | --- | --- | --- | --- | --- | --- |
| *H. perlevis* 1A T24 | 59605 | 55561 | 51657 | 51685 | 43506 | 42516 |
| *H. perlevis* 1B T24 | 124351 | 116557 | 110116 | 109891 | 96079 | 93052 |
| *H. perlevis* 1C T24 | 131647 | 123651 | 117983 | 117799 | 105001 | 103234 |
| *H. perlevis* 1D T24 | 183639 | 173191 | 166542 | 166391 | 149346 | 146008 |
| *H. perlevis* 1E T24 | 81938 | 76833 | 71465 | 71497 | 61394 | 60062 |
| *H. perlevis* 2A T15 | 50770 | 40740 | 35976 | 34314 | 27315 | 26710 |
| *H. perlevis* 2B T15 | 121347 | 95583 | 89559 | 86268 | 70318 | 69257 |
| *H. perlevis* 2C T15 | 86011 | 68110 | 63756 | 61813 | 49770 | 49015 |
| *H. perlevis* 2D T15 | 247266 | 192737 | 185821 | 182215 | 148036 | 143304 |
| *H. perlevis* 2E T15 | 39286 | 31587 | 28252 | 26512 | 21616 | 21356 |
| Seawater 1A T24 | 104680 | 98744 | 94299 | 94364 | 84450 | 78174 |
| Seawater 1B T24 | 172688 | 162196 | 151856 | 152145 | 129895 | 122916 |
| Seawater 1C T24 | 64772 | 60556 | 55978 | 56191 | 45685 | 41613 |
| Seawater 1D T24 | 83486 | 78591 | 73256 | 73380 | 62443 | 57657 |
| Seawater 1E T24 | 88091 | 83415 | 78051 | 78236 | 65044 | 58582 |
| Seawater 2A T15 | 167144 | 134994 | 119675 | 115512 | 89245 | 86768 |
| Seawater 2B T15 | 104262 | 77706 | 72646 | 69827 | 53380 | 52305 |
| Seawater 2C T15 | 30072 | 23433 | 16689 | 14797 | 7926 | 7558 |
| Seawater 2D T15 | 114693 | 86965 | 69600 | 63635 | 37819 | 36527 |
| Seawater 2E T15 | 67521 | 55172 | 46555 | 44602 | 33443 | 32835 |
| *S. massa* 1A T22 | 118398 | 91685 | 87072 | 85021 | 68373 | 66437 |
| *S. massa* 1B T22 | 156738 | 126027 | 121855 | 119842 | 99722 | 98881 |
| *S. massa* 1C T22 | 114586 | 91609 | 86030 | 83586 | 64209 | 62761 |
| *S. massa* 1D T22 | 115759 | 93101 | 88695 | 86485 | 69681 | 69159 |
| *S. massa* 1E T22 | 311276 | 238813 | 231641 | 228349 | 177568 | 176119 |
| *S. massa* 2A T15 | 117109 | 110578 | 106900 | 105537 | 97906 | 94253 |
| *S. massa* 2B T15 | 179116 | 168193 | 162878 | 162419 | 146175 | 140893 |
| *S. massa* 2C T15 | 251845 | 237224 | 227832 | 229068 | 200903 | 194938 |
| *S. massa* 2D T15 | 31899 | 29786 | 28071 | 27808 | 24967 | 24848 |
| *S. massa* 2E T15 | 107277 | 101056 | 97472 | 96642 | 87814 | 85694 |
| Seawater 1A T22 | 147615 | 118099 | 111453 | 109215 | 89167 | 87178 |
| Seawater 1B T22 | 96204 | 76402 | 69717 | 67754 | 54676 | 52570 |
| Seawater 1C T22 | 168955 | 124946 | 116805 | 112678 | 84731 | 82884 |
| Seawater 1D T22 | 33329 | 26592 | 23950 | 22618 | 17520 | 17315 |
| Seawater 1E T22 | 116766 | 94329 | 75873 | 71642 | 43066 | 41860 |
| Seawater 2A T15 | 167308 | 157770 | 147682 | 147820 | 123802 | 114841 |
| Seawater 2B T15 | 36684 | 34600 | 28866 | 29048 | 17441 | 16965 |
| Seawater 2C T15 | 50647 | 47857 | 42102 | 42135 | 31826 | 30467 |
| Seawater 2D T15 | 124432 | 117264 | 107867 | 108021 | 84593 | 79246 |
| Seawater 2E T15 | 92436 | 87376 | 80272 | 80663 | 64286 | 59276 |
| Negative | 29 | 15 | 1 | 2 | 0 | 0 |

**Table S2.** Alpha diversity matrices values (Observed, Shannon, Simpson) for A) *H. perlevis* and *S. massa*, B) *H. perlevis* and associated seawater and C) *S. massa* and associated seawater (T24= temperature 24 °C, T22= temperature 22 °C, T15= temperature 15 °C)

| Group | Measure | N | Mean | SD | SE |
| --- | --- | --- | --- | --- | --- |
| A |  |  |  |  |  |
| *H. perlevis* T24 | Observed | 5 | 991 | 271.55 | 121.44 |
| *H. perlevis* T24 | Shannon | 5 | 5.12 | 0.23 | 0.11 |
| *H. perlevis* T24 | Simpson | 5 | 0.97 | 0.01 | 0.003 |
| *H. perlevis* T15 | Observed | 5 | 479.6 | 285.73 | 127.78 |
| *H. perlevis* T15 | Shannon | 5 | 4.35 | 0.48 | 0.22 |
| *H. perlevis* T15 | Simpson | 5 | 0.95 | 0.02 | 0.01 |
| *S. massa* T22 | Observed | 5 | 513.8 | 256.38 | 114.66 |
| *S. massa* T22 | Shannon | 5 | 2.95 | 0.28 | 0.12 |
| *S. massa* T22 | Simpson | 5 | 0.81 | 0.02 | 0.01 |
| *S. massa* T15 | Observed | 5 | 570.2 | 183.18 | 81.92 |
| *S. massa* T15 | Shannon | 5 | 3.66 | 1.09 | 0.49 |
| *S. massa* T15 | Simpson | 5 | 0.86 | 0.07 | 0.03 |
|  |  |  |  |  |  |
| B |  |  |  |  |  |
| *H. perlevis* T24 | Observed | 5 | 936.4 | 192.06 | 85.89 |
| *H. perlevis* T24 | Shannon | 5 | 5.32 | 0.21 | 0.09 |
| *H. perlevis* T24 | Simpson | 5 | 0.98 | 0.01 | 0.003 |
| *H. perlevis* T15 | Observed | 5 | 600.6 | 324.06 | 144.92 |
| *H. perlevis* T15 | Shannon | 5 | 4.83 | 0.47 | 0.21 |
| *H. perlevis* T15 | Simpson | 5 | 0.96 | 0.02 | 0.01 |
| Water T24 | Observed | 5 | 462.8 | 185.89 | 83.13 |
| Water T24 | Shannon | 5 | 5.12 | 0.26 | 0.12 |
| Water T24 | Simpson | 5 | 0.99 | 0.002 | 0.0008 |
| Water T15 | Observed | 5 | 578 | 247.74 | 110.79 |
| Water T15 | Shannon | 5 | 5.44 | 0.47 | 0.21 |
| Water T15 | Simpson | 5 | 0.99 | 0.005 | 0.002 |
|  |  |  |  |  |  |
| C |  |  |  |  |  |
| *S. massa* T22 | Observed | 5 | 526.4 | 262.92 | 117.58 |
| *S. massa* T22 | Shannon | 5 | 3.03 | 0.34 | 0.15 |
| *S. massa* T22 | Simpson | 5 | 0.82 | 0.02 | 0.01 |
| *S. massa* T15 | Observed | 5 | 741.4 | 256.6 | 114.76 |
| *S. massa* T15 | Shannon | 5 | 3.89 | 1.15 | 0.52 |
| *S. massa* T15 | Simpson | 5 | 0.87 | 0.07 | 0.03 |
| Water T22 | Observed | 5 | 657.6 | 245.37 | 109.73 |
| Water T22 | Shannon | 5 | 5.33 | 0.23 | 0.1 |
| Water T22 | Simpson | 5 | 0.99 | 0.003 | 0.001 |
| Water T15 | Observed | 5 | 980.4 | 344.89 | 154.24 |
| Water T15 | Shannon | 5 | 5.95 | 0.42 | 0.19 |
| Water T15 | Simpson | 5 | 0.99 | 0.006 | 0.003 |

**Table S3.** Alpha diversity statistics (Observed, Shannon, Simpson) for A) *H. perlevis* and *S. massa*, B) *H. perlevis* and associated seawater and C) *S. massa* and associated seawater measured by Kruskal-Wallis one-way ANOVA (*= p≤0.05, **= p≤0.01, ns= not significant) (T24= temperature 24 °C, T22= temperature 22 °C, T15= temperature 15 °C)

| Comparison | Measure | Group | P value unadjusted | P value adjusted | Significance |
| --- | --- | --- | --- | --- | --- |
| A |  |  |  |  |  |
| *H. perlevis* T24 - *H. perlevis* 15 - *S. massa* T22 - *S. massa* T15 | Observed | *H. perlevis* T24 | 0.068 | 0.07 | ns |
| *H. perlevis* T24 - *H. perlevis* T15 - *S. massa* T22 - *S. massa* T15 | Shannon | *H. perlevis* T24 | 0.005 | 0.012 | * |
| *H. perlevis* T24 - *H. perlevis* T15 - *S. massa* T22 - *S. massa* T15 | Simpson | *H. perlevis* T24 | 0.002 | 0.008 | ** |
|  |  |  |  |  |  |
| B |  |  |  |  |  |
| *H. perlevis* T24 - *H. perlevis* T15 - Water T24 - Water T15 | Observed | *H. perlevis* T24 | 0.1 | 0.14 | ns |
| *H. perlevis* T24 - *H. perlevis* T15 - Water T24 - Water T15 | Shannon | Water T15 | 0.17 | 0.17 | ns |
| *H. perlevis* T24 - *H. perlevis* T15 - Water T24 - Water T15 | Simpson | Water T24 | 0.004 | 0.014 | * |
|  |  |  |  |  |  |
| C |  |  |  |  |  |
| *S. massa* T22 - *S. massa* T15 - Water T22 - Water T15 | Observed | Water T15 | 0.17 | 0.24 | ns |
| *S. massa* T22 - *S. massa* T15 - Water T22 - Water T15 | Shannon | Water T15 | 0.003 | 0.006 | ** |
| *S. massa* T22 - *S. massa* T15 - Water T22 - Water T15 | Simpson | Water T15 | 0.002 | 0.006 | ** |

**Table S4.** Permutational multivariate analysis of variance (PERMANOVA) values (based on 999 permutations) for *H. perlevis, S. massa* and associated seawater (T24= temperature 24 °C, T22= temperature 22 °C, T15= temperature 15 °C)

| A |  | | | | |  | | | | | | | | | | | |  | | | | | | |
| --- | --- | --- | --- | --- | --- | --- | --- | --- | --- | --- | --- | --- | --- | --- | --- | --- | --- | --- | --- | --- | --- | --- | --- | --- |
|  | ***H.perlevis* T24** | | | | | ***H.perlevis* T15** | | | | | | | | | | | | ***S. massa* T22** | | | | | | |
| *H.perlevis* T15 | R2=0.289 | | F=3.264 | | P=0.011 |  | | | | | | | | | | | |  | | | | | | |
| *S. massa* T22 | R2=0.818 | | F=35.921 | | P=0.008 | R2=0.805 | | | | | F=33.074 | | | | | | P=0.005 |  | | | | | | |
| *S. massa* T15 | R2=0.6997 | | F=18.635 | | P=0.008 | R2= 0.7 | | | | | | F=18.738 | | | | | P=0.006 | R2=0.326 | | | F=3.843 | | | P=0.005 |
| B |  | | | | |  | | | | | | | | | | | |  | | | | | | |
|  | ***H.perlevis* T24** | | | | | ***H.perlevis* T15** | | | | | | | | | | | | **Seawater T24** | | | | | | |
| H.perlevis T15 | R2=0.249 | F=2.65 | | P=0.011 | |  | | | | | | | | | | | |  | | | | | | |
| Seawater T24 | R2=0.79 | F=29.933 | | P=0.006 | | R2=0.741 | | | | F=0.741 | | | | | | P=0.011 | |  | | | | | | |
| Seawater T15 | R2=0.488 | F=6.66 | | P=0.006 | | R2=0.472 | | | F=6.257 | | | | | | P=0.007 | | | R2=0.36 | F=3.924 | | | | P=0.01 | |
| C |  | | | | |  | | | | | | | | | | | |  | | | | | | |
|  | ***S. massa* T22** | | | | | ***S. massa* T15** | | | | | | | | | | | | **Seawater T22** | | | | | | |
| S. massa T15 | R2=0.323 | F=3.812 | | P=0.008 | |  | | | | | | | | | | | |  | | | | | | |
| Seawater T22 | R2=0.813 | F=34.782 | | P=0.009 | | R2=0.696 | | F=18.323 | | | | | | P=0.009 | | | |  | | | | | | |
| SeawaterT 15 | R2=0.623 | F=13.231 | | P=0.013 | | R2=0.509 | F=8.307 | | | | | | P=0.015 | | | | | R2=0.476 | | F=7.255 | | P=0.011 | | |

**Table S5.** Mean relative abundance counts at taxonomic level of class detected in samples

of *H. perlevis* and *S. massa* at each temperature (samples rarefied to 18,606 reads)

(T24= temperature 24 °C, T22= temperature 22 °C, T15= temperature 15 °C) Standard error for each mean shown in brackets.

| Phylum | Class | Mean relative abundance per group (%) | | | |
| --- | --- | --- | --- | --- | --- |
|  |  | ***H. perlevis* T24** | ***H. perlevis* T15** | ***S. massa* T22** | ***S. massa* T15** |
| *Proteobacteria* | ***Alphaproteobacteria*** | 33.3  (2) | 30  (1.4) | 74.9  (1.7) | 65.6  (6.1) |
|  | ***Gammaproteobacteria*** | 28  (1.3) | 37  (2.2) | 7.2  (1) | 11.4  (2.3) |
| *Bacteroidota* | ***Bacteroidia*** | 14  (1.8) | 8.8  (2) | 8.9  (1.3) | 11.4  (1.9) |
| *Planctomycetota* | ***Planctomycetes*** | 2.7  (0.5) | 2  (0.7) | 1.4  (0.3) | 2.6  (0.6) |
| *Spirochaetota* | ***Leptospirae*** | 1.7  (0.3) | 3.6  (1.7) | 0 | 0 |
| *Actinobacteriota* | ***Acidimicrobiia*** | 2  (0.4) | 1.7  (0.5) | 0.5  (0.09) | 1.6  (0.6) |
| *Dadabacteria* | ***Dadabacteriia*** | 0.6  (0.3) | 1  (0.2) | 0.05  (0.01) | 0.5  (0.2) |
| *Cyanobacteria* | ***Cyanobacteriia*** | 0.7  (0.2) | 0.6  (0.3) | 1.7  (0.4) | 0.2  (0.08) |
| *Patescibacteria* | ***Parcubacteria*** | 2  (0.2) | 0.6  (0.1) | 2.3  (0.6) | 0.4  (0.3) |

**Table S6.** Mean relative abundance counts at taxonomic level of class detected in samples of *H. perlevis* and associated seawater (samples rarefied to 9,096 reads) (T24= temperature 24 °C, T15= temperature 15 °C) Standard error for each mean shown in brackets.

| Phylum | Class | Mean relative abundance per group (%) | | | |
| --- | --- | --- | --- | --- | --- |
|  |  | ***H. perlevis* T24** | ***H. perlevis* T15** | **Seawater**  **T24** | **Seawater**  **T15** |
| *Proteobacteria* | ***Alphaproteobacteria*** | 35.7  (2.1) | 34  (1.5) | 27.9  (1) | 18.1  (3.6) |
|  | ***Gammaproteobacteria*** | 18.7  (13) | 17.2  (1.9) | 5.4  (0.6) | 13  (3.3) |
| *Bacteroidota* | ***Bacteroidia*** | 15.7  (1.9) | 10.8  (2.3) | 59  (1.7) | 38.6  (5.7) |
| *Planctomycetota* | ***Planctomycetes*** | 3.3  (0.6) | 2.9  (0.9) | 0.4  (0.1) | 5.3  (4) |
| *Spirochaetota* | ***Leptospirae*** | 1.8  (0.3) | 3.6  (1.4) | 0 | 0 |
| *Actinobacteriota* | ***Acidimicrobiia*** | 2  (0.4) | 2.3  (0.5) | 0.2  (0.04) | 0.9  (0.3) |
| *Dadabacteria* | ***Dadabacteriia*** | 0.59  (0.3) | 1.2  (0.3) | 0 | 0 |
| *Cyanobacteria* | ***Cyanobacteriia*** | 0.7  (0.2) | 0.7  (0.3) | 0.08  (0.01) | 0.6  (0.2) |
| *Patescibacteria* | ***Parcubacteria*** | 2.4  (0.2) | 0.7  (0.1) | 0.6  (0.07) | 3.1  (1.25) |

**Table S7.** Mean relative abundance counts at taxonomic level of class detected in samples of *S. massa* and associated seawater (samples rarefied to 16,664 reads) (T22= temperature 22 °C, T15= temperature 15 °C) Standard error for each mean shown in brackets.

| Phylum | Class | Mean relative abundance per group (%) | | | |
| --- | --- | --- | --- | --- | --- |
|  |  | ***S. massa* T22** | ***S. massa* T15** | **Seawater**  **T22** | **Seawater**  **T15** |
| *Proteobacteria* | ***Alphaproteobacteria*** | 74.3  (1.9) | 63  (6.4) | 17.4  (1.5) | 18.5  (3.1) |
|  | ***Gammaproteobacteria*** | 7.4  (1.1) | 7.5  (1.1) | 11.2  (1.9) | 12.3  (1.6) |
| *Bacteroidota* | ***Bacteroidia*** | 8.8  (1.2) | 8.8  (1.2) | 52.7  (5.4) | 20.4  (3.6) |
| *Planctomycetota* | ***Planctomycetes*** | 1.1  (0.2) | 0.6  (0.2) | 2.8  (0.8) | 19.9  (5) |
| *Actinobacteriota* | ***Acidimicrobiia*** | 0.48  (0.1) | 0.5  (0.1) | 1.3  (0.4) | 0.8  (0.1) |
| *Cyanobacteria* | ***Cyanobacteriia*** | 1.7  (0.4) | 1.7  (0.5) | 0.1  (0.04) | 0.2  (0.1) |
| *Patescibacteria* | ***Parcubacteria*** | 2.4  (0.7) | 2.4  (0.7) | 0.5  (0.2) | 3.6  (1.6) |
| *Nitrospirota* | ***Nitrospiria*** | 0.2  (0.1) | 0.2  (0.1) | 0.002  (0.002) | 0.01  (0.005) |

**Table S8.** Mean relative abundance counts at taxonomic level of family detected in samples of *H. perlevis* and *S. massa* (samples rarefied to 18,606 reads) (T24= temperature 24 °C, T22= temperature 22 °C, T15= temperature 15 °C) Standard error for each mean shown in brackets.

| Family | Mean relative abundance per group (%) | | | |
| --- | --- | --- | --- | --- |
|  | ***H. perlevis* T24** | ***H. perlevis* T15** | ***S. massa* T22** | ***S. massa* T22** |
| *Flavobacteriaceae* | 10.2  (1.7) | 7.7  (1.7) | 7.4  (1.2) | 7.7  (1.4) |
| *Terasakiellaceae* | 18.6  (1.9) | 17.1  (1.9) | 67.2  (1.4) | 57.3  (7.9) |
| SAR 116 | 2.6  (0.6) | 3.3  (1.2) | - | - |
| *Leptospiraceae* | 1.6  (0.3) | 3.6  (1.6) | - | - |
| *Sphingomonadaceae* | 0.5  (0.1) | 0.3  (0.2) | - | - |
| *Cyclobacteriaceae* | 0.6  (0.2) | 0.3  (0.4) | 0.7  (0.2) | 2.2  (0.2) |
| *Cyanobiaceae* | 0.6  (0.2) | 0.4  (0.05) | 1.8  (0.3) | 0.2  (0.08) |

**Table S9.** Mean relative abundance counts at taxonomic level of family detected in samples of *H. perlevis* and associated seawater (samples rarefied to 9,096 reads) (T24= temperature 24 °C, T15= temperature 15 °C) Standard error for each mean shown in brackets.

| Family | Mean relative abundance per group (%) | | | |
| --- | --- | --- | --- | --- |
|  | ***H. perlevis* T24** | ***H. perlevis* T15** | **Water T24** | **Water T15** |
| *Flavobacteriaceae* | 11.3  (1.9) | 9  (2.3) | 27.9  (1.4) | 15  (5.2) |
| *Terasakiellaceae* | 20  (2) | 18  (1.9) | - | - |
| *Rhodobacteraceae* | 4.2  (0.4) | 3.9  (0.5) | 15.8  (1) | 12.5  (3.8) |
| *Leptospiraceae* | 1.9  (0.3) | 3.7  (1.6) | - | - |
| SAR 116 | 2.6  (0.5) | 3.7  (1.4) | 2.9  (0.2) | 1.1  (0.4) |
| *Sphingomonadaceae* | 0.5  (0.1) | 0.3  (0.2) | - | - |
| *Cryomorphaceae* | 0.5  (0.2) | 0.3  (0.2) | 7  (0.3) | 4.4  (1.5) |
| Unidentified family (Order: Dadabacteriales) | 0.7  (0.2) | 1.2  (0.2) | - | - |

**Table S10.** Mean relative abundance counts at taxonomic level of family detected in samples of *S. massa* and associated seawater (samples rarefied to 16,664 reads) (T22= temperature 22 °C, T15= temperature 15 °C) Standard error for each mean shown in brackets.

| Family | Mean relative abundance per group (%) | | | |
| --- | --- | --- | --- | --- |
|  | ***S. massa* T22** | ***S. massa* T22** | **Water T22** | **Water T15** |
| *Flavobacteriaceae* | 7.2  (1) | 7.6  (1.4) | 40.4  (4.2) | 10.6  (2.4) |
| *Terasakiellaceae* | 66.6  (1.4) | 54.9  (8) | - | - |
| *Cyclobacteriaceae* | 0.5  (0.2) | 2.4  (0.6) | 07.7  (0.9) | 1.4  (0.3) |
| *Cyanobiaceae* | 1.8  (0.5) | 0.2  (0.1) | 0.1  (0.03) | 0.1  (0.03) |
| *Rhodobacteraceae* | 0.83  (0.3) | 2.7  (0.8) | 12.2  (1) | 4.1  (0.2) |
| *Pirellulaceae* | 1.4  (0.3) | 3.2  (0.4) | 1.7  (0.6) | 3.8  (0.6) |
